# Influencing Factors of Psychological Well-Being of the Non-designated Hospital Staff in China During the COVID-19 Pandemic

**DOI:** 10.3389/fpsyt.2021.591026

**Published:** 2021-02-04

**Authors:** Dandan Yao, Jihui Lyu, Zongjuan Ma, Mei Champ, Qian Xiong, Mo Li, Wenjie Li, Haiyan Mu, Yueqing Hu, Wenchao Gao, Dongmei Jia

**Affiliations:** ^1^Center for Cognitive Disorders, Beijing Geriatric Hospital, Beijing, China; ^2^Beijing Institute of Brain Disorders, Laboratory of Brain Disorders, Ministry of Science and Technology, Collaborative Innovation Center for Brain Disorders, Capital Medical University, Beijing, China; ^3^Department of Nursing and Midwifery, University of the West of England, Bristol, United Kingdom; ^4^Centre for Ageing Research (C4AR), Division of Health Research, Faculty of Health and Medicine, Lancaster University, Lancaster, United Kingdom

**Keywords:** COVID-19, psychological well-being, non-designated hospital staff, social support, coping style

## Abstract

**Background:** Recent studies report that hospital staff at the forefront of caring for COVID-19 patients experience increased psychological distress. To effectively manage the outbreak of COVID-19, China established COVID-19 designated and non-designated hospitals. To date, few studies have examined the impacts of COVID-19 on psychological health of staff working at non-designated hospitals. This study is to explore factors affecting psychological health of non-designated hospital staff in China during the COVID-19 pandemic.

**Methods:** Data were collected through an online questionnaire between February and March 2020. The questionnaire consists of General Health Questionnaire (GHQ-20), Social Support Rating Scale (SSRS), Simplified Coping Style Questionnaire (SCSQ), sociodemographic characteristics, employment history, health status, and contact history of COVID-19. The questionnaire was distributed through hospital WeChat groups and work colleague referrals. A total of 470 non-designated hospital staff members completed the questionnaire. Multiple Linear Regression analysis was used to interpret the associations among social support, coping styles, sociodemographic factors, job roles, and psychological status. Data were analyzed using SPSS version 21.0.

**Results:** The non-designated hospital staff differed significantly in anxiety and depression subscores of the GHQ-20 by their job roles, levels of social support, and history of mental disorders. Staff with medical job roles, good self-reported health status, no previous mental disorders, adequate social support, and positive coping styles scored lower in GHQ-20 total score, which indicated healthier psychological status.

**Conclusions:** The results indicate that history of mental health disorders, non-medical job roles, and inadequate social support are associated with greater psychological distress. Personalized support should be provided to those who are vulnerable and in need of social and psychological support.

## Introduction

On January 30th, 2020, the World Health Organization (WHO) declared an outbreak of a public health emergency of international concern. COVID-19 is the third coronavirus pneumonia which has induced a global pandemic after SARS and MERS in the 21st century (1–3). By January 3rd, 2021, a total number of 83.33 million confirmed COVID-19 cases and 1.83 million deaths were declared by WHO, and the number of deaths is likely to increase as the pandemic continues (4). In order to control the spread of the COVID-19 infection and maintain the routine treatment for patients, COVID-19 designated hospitals (DHs) were set up to manage patients who had been diagnosed with COVID-19 in China, while other hospitals were non-designated hospitals (NDHs).

Serious contagious public health events can lead to a series of stress reactions. COVID-19 has caused serious physical medical conditions as well as psychological distress, tension, and anxiety among healthcare workers (5–9). The psychological stress that the hospital staff experience in a high-risk and high-pressured environment may affect work efficiency and clinical outcomes (10). During the pandemic, hospital staff either working in COVID-19 DHs or NDHs have all experienced increased psychological stress (11). The NDH staff not only encounter the challenges of screening suspected cases of this highly contagious disease, but are also at risk of contracting COVID-19 themselves (12, 13). However, only few studies focused on psychological well-being of NDH staff. It is important to investigate the potential risk factors so that appropriate health and safety measures could be implemented to improve the psychological health of the NDH staff. Therefore, this study aims to explore factors affecting psychological well-being of NDH staff during the COVID-19 pandemic.

## Methods

### Study Design and Participants

This research is a cross-sectional study using convenience and snowballing sampling approach. An online survey was selected due to its low cost, convenience, and real time access. It allows participants to complete the survey based on their own schedule and at their own pace (14–16).

Questionnaire Star, an application of survey, was used to develop the online electronic questionnaire. The questionnaire was distributed to the hospital staff via six nationwide WeChat hospital groups. WeChat is a widely used social media platform in China. The eligibility of the WeChat members was assessed by the group managers and only verified hospital staff including doctors, nurses, administrative and logistic personnel could be accepted. All staff members working at NDHs within the WeChat groups were invited to complete the questionnaire voluntarily and anonymously. Participants were also encouraged to recommend their hospital work colleagues, who met the criteria, to participate in this study. Data were collected between February 20th and March 20th, 2020. All questions were mandatory to be completed by participants; only fully completed questionnaire could be submitted and accepted. The questionnaire was purposely designed to automatically lock its editable function and accessibility once submitted.

Data collected through the questionnaire included sociodemographic characteristics, employment history, health status, contact history of COVID-19, psychological status, social support, and coping style of the participants. The questionnaire consists of five main sections.

#### Sociodemographic, Employment, and Health Data Collection

Sociodemographic characteristics included age, gender, length of employment, educational level (Diploma, Foundation Degree, Bachelor, Masters, and Doctoral), marital status (single, married, divorced, or widowed), and job roles (medical staff including doctors, nurses, and medical technicians; non-medical staff including administrative and logistical personnel). The staff were graded into junior, middle, and senior tiers according to the length of employment and qualification levels in accordance with the hospital staff grading system in China. The departments were categorized as the reception, fever clinic, emergency department, respiratory department, other medical departments (neurology and gastroenterology), surgical departments, and other departments (intensive care unit, medical laboratory, administrative, and logistics). Self-reported health status was rated as good, fair, or poor. Participants were also asked whether they had any underlying medical conditions such as diabetes, hypertension, immune disorders, and other physical conditions including neoplastic disease and coronary heart disease; whether they had a history of mental disorders of depression, anxiety, insomnia, obsessive-compulsive disorder, or other mental disorders.

#### Contact History of COVID-19

The information of whether the participants had been in contact with relatives or friends, who were confirmed or suspected of COVID-19 and whether they had been quarantined, were collected.

#### Psychological Status

The General Health Questionnaire (GHQ-20) compiled by Goldberg (17) and revised by Li et al. (18) was used to assess psychological status. There are 20 items in total, including nine items for self-affirmation subscale, 6 items for depression subscale (scores 6–24), and five items for anxiety subscale (scores 5–20). The items were scored ranging from 1 (never) to 4 (often). Self-affirmation items were reversely scored to generate a self-affirmation subscore between 9 and 36, the higher the self-affirmation subscore, the lower level of self-affirmation. The higher the depression or anxiety subscore indicates the greater degree of depression or anxiety. The sum of self-affirmation, depression, and anxiety subscores is the GHQ total score, ranging from 20 to 80, the higher the GHQ total score, the poorer psychological health of the individuals. There is no cut-off point for “case” identification in this Chinese version of GHQ-20 according to previous studies (18, 19).

#### Social Support

Social support was measured by the Social Support Rating Scale (SSRS) (20). There are 10 SSRS items classified into three dimensions. These dimensions are objective support (items 2, 6, 7), subjective support (items 1, 3, 4, 5), and utilization of support (items 8, 9, 10). Items 1–4 and 8–10 are scored on a four-point scale (none = 1, little = 2, some = 3, and full = 4). Item five for social support received from family members has five categories (spouse, parents, children, siblings, or other family members). Each category is scored on a four-point scale (none = 1, little = 2, some = 3 or full = 4). The score of each category contributes to social support level. Item 6 and 7 are scored ranging from “have no source = 0” to “have all the following sources = 8”. The total score of 10 items ranges from 12 to 64 indicating three levels of social support (12 to 33 = low, 34 to 45 = medium, and 46 to 64 = high). This scale has been widely used and its internal consistency coefficient is reported as 0.92, indicating good reliability (21).

#### Coping Style

The coping style was measured by the Simplified Coping Style Questionnaire (SCSQ), which has good reliability and is commonly used in China (22). The SCSQ is a self-rated questionnaire with 20 items. The score of each item ranges from “never = 0” to “often = 3.” The SCSQ encompasses two dimensions: positive coping style (items 1–12) and negative coping style (items 13–20). The higher the dimension score, the more frequent the corresponding coping style is used by individuals with stress.

### Statistical Analysis

Descriptive analysis was carried out to show the characteristics of the sample. The means (standard deviation) were used to describe the continuous variables. Frequency and percentage were used to describe the categorical variables. Multiple linear regression models were conducted to evaluate the associations among independent variables and dependent variables. The principle of statistical sample size estimation stipulates that number of participants in multiple regression analysis needs to be at least 10 times of the number of independent variables (23). Fifteen independent variables in each multiple regression analysis are presented in this study. The sample size of 470 exceeds 30 times of the number of independent variables. To identify the influencing factors of psychological well-being of NDH staff, the study used the GHQ total score and its subscores as dependent variables, and sociodemographic characteristics, employment history, health status, contact history of COVID-19, social support level, and SCSQ subscores as independent variables. All data were analyzed using IBM SPSS Statistics for Windows, Version 21.0 (Release 2011, IBM Corp, Armonk, NY, USA). A value of *p* < 0.05 is considered statistically significant.

## Results

### Sociodemographic Characteristics, Employment History, Health Status, and Contact History of COVID-19

A total number of 470 complete questionnaires were received. The demographic characteristics of the participants are shown in Table 1. Among 470 participants, 388 were medical staff (219 nurses, 86 doctors, and 83 medical technicians) and 82 were non-medical staff (31 administrative staff and 51 logistical personnel). One hundred and three participants self-reported with underlying medical conditions of diabetes (*n* = 15), hypertension (*n* = 36), immune disorders (*n* = 8), and other physical conditions (*n* = 44). Seventy participants had a history of mental disorders. These were depression (*n* = 10), anxiety (*n* = 12), insomnia (*n* = 38), obsessive-compulsive disorder (*n* = 5), and other mental disorders (*n* = 5). The results from participants who self-rated their health illustrated the following: 299 (63.61%) good, 154 (32.77%) fair, and 17 (3.62%) poor.

**Table 1:** Sociodemographic characteristics of participants, mean value and SD of GHQ total score, self-affirmation subscore, anxiety subscore, and depression subscore.

**Variables**		**GHQ total score**	**Self-affirmation subscore (Reversely coded)**	**Anxiety subscore**	**Depression subscore**
	***N* (%)**	**Mean (SD)**	**Mean (SD)**	**Mean (SD)**	**Mean (SD)**
Total	470 (100.00)	33.16 (9.70)	13.84 (3.74)	9,62 (3.60)	9.71 (3.66)
**Gender**					
Male	101 (21.49)	33.73 (9.28)	14.04 (3.53)	9.95 (3.61)	9.74 (3.43)
Female	369 (78.51)	33.01 (9.81)	13.78 (3.80)	9.53 (3.60)	9.70 (3.73)
**Marital status**					
Single	91 (19.36)	31.48 (9.49)	14.16 (4.07)	8.42 (3.29)	8.90 (3.56)
Married	364 (77.45)	33.25 (9.49)	13.64 (3.58)	9.83 (3.57)	9.79 (3.60)
Divorced	12 (2.55)	42.92 (11.46)	17.25 (4.16)	12.58 (3.83)	13.08 (4.10)
Widowed	3 (0.64)	34.00 (14.73)	14.67 (4.51)	8.67 (5.51)	10.67 (5.69)
**Educational level**					
Diploma	21 (4.47)	32.43 (6.80)	12.90 (2.34)	9.90 (3.85)	9.62 (3.22)
Foundation Degree	101 (21.49)	32.79 (10.05)	13.72 (3.60)	9.48 (3.69)	9.59 (3.86)
Bachelor	272 (57.87)	33.21 (9.86)	13.93 (3.89)	9.57 (3.68)	9.71 (3.59)
Masters	64 (13.62)	34.08 (9.66)	14.03 (3.73)	9.98 (3.23)	10.06 (3.94)
Doctoral	12 (2.55)	31.50 (8.22)	13.42 (3.55)	9.33 (2.84)	8.75 (3.17)
**Staff grade**					
Junior	228 (48.51)	32.37 (10.24)	14.07 (4.10)	8.95 (3.63)	9.36 (3.78)
Middle	152 (32.34)	33.74 (9.08)	13.63 (3.34)	10.13 (3.40)	9.98 (3.60)
Senior	90 (19.15)	34.19 (9.23)	13.61 (3.38)	10.44 (3.59)	10.13 (3.41)
**Job role**					
Medical staff^a^	388 (82.6)	32.81 (9.743)	13.82 (3.70)	9.43 (3.60)	9.57 (3.65)
Non-medical staff^b^	82 (17.4)	34.82 (9.36)	13.93 (3.93)	10.52 (3.48)	10.37 (3.66)
**Department**					
Reception desk	12 (2.55)	29.08 (7.37)	11.75 (2.30)	9.00 (3.02)	8.33 (2.71)
Fever clinic	25 (5.32)	30.96 (10.32)	13.20 (3.67)	8.88 (3.69)	8.88 (3.61)
Emergency	16 (3.40)	31.56 (7.15)	13.31 (3.79)	9.13 (2.90)	9.13 (2.22)
Respiratory department	24 (5.11)	28.04 (6.52)	12.42 (2.65)	7.63 (2.93)	8.00 (2.49)
Other medical departments	116 (24.68)	32.63 (9.88)	13.71 (3.59)	9.34 (3.65)	9.58 (3.70)
Surgical department	39 (8.30)	31.33 (7.71)	13.13 (2.99)	9.13 (3.05)	9.08 (3.01)
Other	238 (50.64)	34.78 (10.05)	14.37 (3.99)	10.17 (3.70)	10.24 (3.88)
**Self-reported health status**					
Poor	17 (3.62)	44.18 (13.90)	18.41 (7.08)	12.82 (4.52)	12.94 (4.58)
Fair	154 (32.77)	37.36 (10.53)	15.23 (3.87)	10.94 (3.79)	11.19 (4.15)
Good	299 (63.61)	30.37 (7.54)	12.86 (2.91)	8.75 (3.12)	8.76 (2.92)
**Underlying medical condition**					
No	367 (78.09)	31.57 (8.53)	13.41 (3.31)	9.02 (3.25)	9.15 (3.33)
Yes^c^	103 (21.91)	38.83 (11.40)	15.37 (4.68)	11.76 (3.96)	11.70 (4.11)
**A history of mental disorder**					
No	400 (85.11)	31.15 (7.94)	13.19 (3.08)	8.94 (3.15)	9.02 (3.09)
Yes^d^	70 (14.89)	44.66 (10.84)	17.54 (4.88)	13.47 (3.61)	13.64 (4.21)
**Relative or friend confirmed or suspected with COVID-19**					
No	464 (98.72)	33.22 (9.73)	13.86 (3.75)	9.64 (3.60)	9.72 (3.66)
Yes	6 (1.28)	28.50 (5.54)	11.83 (2.14)	7.67 (3.33)	9.00 (4.15)
**Placed in quarantine**					
No	427 (90.85)	33.19 (9.85)	13.81 (3.83)	9.66 (3.61)	9.72 (3.68)
Yes	43 (9.15)	32.93 (8.18)	14.12 (2.67)	9.21 (3.54)	9.60 (3.59)
Age (years), mean (range)	37.10 (20-62)	–	–	–	–
Length of employment (years), mean (range)	15.10 (0-45)	–	–	–	–

*GHQ, general health questionnaire; SD, standard deviation; COVID-19, coronavirus disease 2019*.

a *Medical staff included doctor, nurse, and medical technician*.

b *Non-medical staff included administrative personnel and logistical personnel*.

c *Chronic medical condition included diabetes, hypertension, immunological disease and other*.

d *Mental disorder included depression, anxiety, insomnia, obsessive-compulsive disorder, and other*.

### Influencing Factors of Psychological Well-Being

Four multiple linear regression analyses were conducted to detect the factors that may impact on psychological well-being. Table 2 reports the results of multiple regressions with variables that had significant coefficients for at least one of the outcomes. The results of the regression model evaluation are listed in Table 2. F test results of the four models were 16.972, 12.306, 11.161, and 12.312, respectively. All *p*-values were <0.001, suggesting that the independent variables predicted the dependent variables. Durbin-Watson values (1.886, 1.971, 1.845, and 1.891) supported that there was no first-order linear autocorrelation in the four multiple linear regressions data.

**Table 2:** Variables that explain variance in GHQ total score, self-affirmation subscore, anxiety subscore, and depression subscore of hospital staff.

**Variables**	**GHQ total score**			**Self-affirmation subscore**			**Anxiety subscore**			**Depression subscore**		
	**β**	***t***	***p*-value**	**β**	***t***	***p*-value**	**β**	***t***	***p*-value**	**β**	***t***	***p*-value**
Intercept		8.820	<0.001		9.601	<0.001		6.046	<0.001		5.723	<0.001
**Job role (Non-medical staff^b^ = Reference)**												
Medical staff^a^	−0.083	−2.077	0.038	−0.002	−0.039	0.969	−0.127	−2.861	0.004	−0.094	−2.172	0.030
**Self-reported health status (Good = Reference)**												
Poor	0.062	1.658	0.098	0.135	3.312	0.001	0.014	0.338	0.735	0.014	0.339	0.735
fair	0.099	2.554	0.011	0.140	3.342	0.001	0.042	0.983	0.326	0.078	1.855	0.064
**A history of mental disorder^c^ (No = Reference)**												
Yes	0.259	6.707	<0.001	0.189	4.528	<0.001	0.256	5.989	<0.001	0.242	5.784	<0.001
**Social support level (High = Reference)**												
Low	0.176	3.747	<0.001	0.157	3.102	0.002	0.109	2.105	0.036	0.197	3.893	<0.001
Medium	0.113	2.680	0.008	0.140	3.059	0.002	0.039	0.843	0.400	0.118	2.592	0.010
**Coping style scores**												
Positive	−0.363	−10.289	<0.001	−0.336	−7.784	<0.001	−0.320	−7.247	<0.001	−0.305	−7.053	<0.001
Negative	0.226	6.475	<0.001	0.143	3.789	<0.001	0.212	5.518	<0.001	0.243	6.442	<0.001
R^2^	0.528			0.448			0.424			0.448		
F	16.972^*^			12.306^*^			11.161^*^			12.312^*^		
Durbin-Watson value	1.886			1.971			1.845			1.891		

*GHQ, general health questionnaire; SSRS, social support sating scale*.

a *Medical staff included doctor, nurse, and medical technician*.

b *Non-medical staff included administrative personnel and logistical personnel*.

c *Mental disorder included depression, anxiety, insomnia, obsessive-compulsive disorder, and other*.

* *p < 0.001*.

The first model accounted for 52.8% of the variance in the GHQ-20 total score. Medical staff (β = −0.083, *p* = 0.038) and positive coping style (β = −0.363, *p* < 0.001) were associated with lower GHQ-20 total score. Fair self-reported health (β = 0.099, *p* = 0.011), history of mental disorder (β = 0.259, *p* < 0.001), low (β = 0.176, *p* < 0.001) or medium social support level (β = 0.113, *p* = 0.008), and negative coping style (β = 0.226, *p* < 0.001) were associated significantly with higher GHQ-20 total score. Therefore, non-medical staff and participants with fair self-reported health status, a history of mental disorder, lower social support, or more negative coping style, had poorer psychological health outcomes.

The second model explained 44.8% of the variance in the self-affirmation subscore. Positive coping styles (β = −0.336, *p* < 0.001) were associated with lower self-affirmation subscore. Fair (β = 0.140, *p* = 0.001) or poor self-reported health (β = 0.135, *p* = 0.001), history of mental disorder (β = 0.189, *p* < 0.001), low (β = 0.157, *p* = 0.002) or medium social support level (β = 0.140, *p* = 0.002), and negative coping style (β = 0.143, *p* < 0.001) were associated with higher self-affirmation subscore. As the self-affirmation was reversely coded, it suggests that poor self-reported health status, history of mental disorders, lower level of social support, or negative coping style predict lower level of self-affirmation.

The third model gave explanations for 42.4% of the variance in the anxiety subscore. Medical staff (β = −0.127, *p* = 0.004) and positive coping style (β = −0.320, *p* < 0.001) were associated with lower anxiety subscore. The history of mental disorder (β = 0.256, *p* < 0.001), low level of social support (β = 0.109, *p* = 0.036), and negative coping style (β = 0.212, *p* < 0.001) were associated with higher anxiety subscore. The final model accounted for 44.8% of the variance in the depression subscore. Medical staff (β = −0.094, *p* = 0.030) and more positive coping style (β = −0.305, *p* < 0.001) were associated with lower depression subscore. The history of mental disorder (β = 0.242, *p* < 0.001), low (β = 0.197, *p* < 0.001) or medium social support level (β = 0.118, *p* = 0.010), and negative coping style (β = 0.243, *p* < 0.001) were associated with higher depression subscore. The results reveal that non-medical staff and participants with a history of mental disorder, low social support level, or negative coping style tend to have higher level of anxiety and depression.

## Discussion

This survey investigated the psychological well-being of NDH staff during the COVID-19 pandemic in China and explored its influencing factors, which may help understand how to best maintain and improve psychological health of NDH staff. The results show that psychological well-being can be affected by job role, health status, history of mental disorders, social support level, and coping style. However, gender, age, marital status, educational level, underlying medical condition, length of employment, department, staff grade, and contact history of COVID-19 are not likely to have an effect on psychological well-being.

This study found that history of mental disorders was associated with high levels of anxiety and depression. The high prevalent psychological problems among hospital staff during pandemics are anxiety and depression (8, 24). High levels of anxiety and depression among individuals with history of mental disorders may be due to the deterioration of their mental health during a pandemic, which has been demonstrated in previous studies (25, 26). The findings of this study suggest that hospital staff with poor physical health tends to have lower level of self-affirmation. It is known that the high self-affirmation can alleviate stress and negative emotions (27–31). It enhances positive emotions such as optimism and self-esteem (27) and promotes psychological well-being (32). Previous studies have found that individuals with poor health status and underlying medical conditions are at risk of developing unfavorable outcomes of psychological well-being in the face of disasters (26). However, no association was found between underlying medical conditions and poor psychological well-being in this study. Poor self-reported physical health may also be a result of the somatization symptoms of mental health (33).

There is a substantial literature documenting that the incidence of mental health problems among medical staff is higher than other non-service occupational groups (34), especially during pandemics (35, 36). However, several studies found that there were no differences in the incidence of anxiety and depression between medical and non-medical staff within hospital environment during the SARS and COVID-19 outbreaks (37, 38). Non-medical staff presented higher levels of anxiety and depression than medical staff in this survey, which is also stated in a previous study (39). Potential factors contributing toward this finding include that medical staff usually have more knowledge and experience in infection control measures, and they are more likely to be equipped with adequate personal protective equipment (PPE) compared to non-medical staff (9, 38–40). Current studies have reported that inadequate PPE, and having less pandemic knowledge and training are associated with higher levels of psychological distress (41, 42). More support should be provided to non-medical staff, including training, standardizing work procedures, psychological counseling, and appropriate PPE to promote physical and mental health during the pandemic.

Results of this study support that the positive coping style contributes to psychological well-being of the hospital staff, which is consistent with the previous studies (43–46). Coping capability is a complex interaction between the individuals and their circumstances. The coping capability can be divided into emotional-oriented coping strategy, approach-oriented coping strategy, and avoidance-oriented coping strategy. In general, the approach-oriented coping strategy is associated with positive psychological health outcomes, therefore it is described as a positive coping style (47). A positive coping approach can help overcome stress and it is an effective psychological mechanism to improve mental resilience (43, 44). Positive coping strategies helped reduce anxiety and depression levels of hospital staff during the SARS outbreak (45, 46). Interventions that decrease the use of maladaptive coping style can also reduce long-term distress (48). However, poor mental health status influences the approaches of coping strategy which could result in a negative effect (49). Social support, as an important coping resource, contributes to developing individual resilience and effective coping skills in adverse circumstances (50, 51). Social support helps ease psychological distress (52–54). Studies show that higher level of social support was correlated with fewer psychological disorders among hospital staff during the SARS outbreak (48). This investigation confirms a significant associated relationship between social support and psychological well-being. Researchers suggest that hospital staff should be given an adequate level of moral support and protective equipment (39, 55). Staff should be provided appropriate training on pandemic awareness and management related to the daily duties and tasks of different job roles. Besides adequate PPE and welfare support, a dedicated helpline and counseling will be beneficial to the staff, who pose a high risk of developing negative emotions and serious mental illnesses (12).

There are two limitations to this study. The first is that the social distancing rules during COVID-19 pandemic made it difficult to conduct a face to face stratified random sampling survey. The data collected through “Questionnaire Star” was self-reported by the participants. This may result in inductive bias. It was not possible to determine response rates. The participants were recruited from 6 WeChat groups as well as through colleague referrals. However, the study targeted the right audience and received a sufficient number of completed questionnaires. A retrospective face-to-face clinical interview could be considered post the COVID-19 outbreak when possible. The second is that as a cross-sectional investigation it lacks follow-ups. The dynamic change of the psychological distress of the hospital staff as the pandemic continuing needs to be further explored. Thus, a further investigation needs to be developed to continue monitoring the long-term psychological effects on NDH staff.

In conclusion, the results of this study suggest that non-medical staff and those who have a history of mental disorders in NDHs are at high risk of developing anxiety and depression. Adequate social support and positive coping strategies can help reduce the psychological stress level and improve mental well-being. During a pandemic, it is essential to provide personalized support to the hospital staff who are vulnerable and in need of social, medical, and psychological support.

## Data Availability Statement

The raw data supporting the conclusions of this article will be made available by the authors, without undue reservation.

## Ethics Statement

The studies involving human participants were reviewed and approved by Medical Ethics Committee of the Beijing Geriatric Hospital. The patients/participants provided their written informed consent to participate in this study.

## Author Contributions

JL supervised this study and is the guarantor. JL and ZM conceived the idea and designed the method. JL and DY carried out the statistical analysis and interpreted the results. QX suggested analytic strategy and commented on the interpretation of results. DY drafted the manuscript. JL, MC, and QX revised the manuscript. All authors participated in the data collection and preparation of this manuscript. All authors approved the submission of this manuscript.

## Conflict of Interest

The authors declare that the research was conducted in the absence of any commercial or financial relationships that could be construed as a potential conflict of interest. The reviewer HW declared a shared affiliation with several of the authors, DY, JL, ZM, ML, WL, HM, YH, to the handling editor at time of review.
